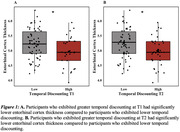# Lower entorhinal cortex thickness is associated with greater temporal discounting in cognitively unimpaired older adults

**DOI:** 10.1002/alz.092719

**Published:** 2025-01-03

**Authors:** Laura E. Fenton, Lauren Salminen, Anya Samek, Gali H Weissberger, Aaron C Lim, Annie Nguyen, Jenna Axelrod, Daisy Noriega‐Makarskyy, Jennifer Herrera, Nathan Wei, Elizabeth Erdman, S. Duke Han

**Affiliations:** ^1^ University of Southern California, Los Angeles, CA USA; ^2^ Imaging Genetics Center, Mark and Mary Stevens Neuroimaging and Informatics Institute, Keck School of Medicine, University of Southern California, Marina del Rey, CA USA; ^3^ University of California San Diego, La Jolla, CA USA; ^4^ Bar Ilan University, Ramat Gan Israel

## Abstract

**Background:**

Alzheimer’s disease (AD) neuropathology may impact brain regions involved in decision making. Because of this, changes in decision making (e.g., economic preferences) may be an early behavioral manifestation of preclinical AD. The current study investigated relationships between temporal discounting (the tendency to choose smaller, more immediate rewards over larger, delayed rewards) and brain structures implicated early in preclinical AD.

**Method:**

The sample included 92 cognitively intact older adults (mean age = 68.8±7.43; mean education = 16.62±2.15; 75% female; 79% White). Temporal discounting was characterized using a paradigm in which participants choose between earlier or later payments of varying amounts. In the first section (T1), participants choose between payments today vs. 2 weeks from today. In the second section (T2), choices were between today vs. 4 weeks from today. In the third section (T3), choices were between 2 weeks vs. 4 weeks from today. Temporal discounting rates were calculated for each time point. Due to most participants clustering around a low modal value of temporal discounting, participants were categorized as “low” on temporal discounting if they were at or below the mode and “high” if they were above the mode. Brain structural MRI estimates were acquired on a 7 Tesla scanner and processed using FreeSurfer‐v.7.2.0. The regions of interest (ROIs) included the entorhinal and parahippocampal cortex and hippocampus. Differences in ROIs between the two groups were tested using one‐way ANOVA models adjusted for age, sex, and education.

**Result:**

Individuals who exhibited higher temporal discounting rates at T1 and T2 had significantly lower entorhinal cortex thickness (F = 6.06, *p* = 0.02 and F = 6.18, *p* = 0.01 respectively). No significant relationship was observed at T3. Additionally, no significant relationships were observed between temporal discounting and parahippocampal cortex thickness or hippocampal volume.

**Conclusion:**

Results showed that lower entorhinal cortex thickness is associated with greater temporal discounting in cognitively intact older adults. This supports the idea that changes in economic preferences may be an early behavioral manifestation of preclinical AD. Due to the cross‐sectional nature of the study, it will be important to validate these findings in longitudinal studies.